# Nocturnal Hypoxemia and Airway Phenotype in Adults with Cancer: An Exploratory Case–Control Study

**DOI:** 10.3390/jcm15156013

**Published:** 2026-08-02

**Authors:** Carlos Mas Bermejo, Carlos Mas Gómez, Luis-Alberto Bravo-González

**Affiliations:** 1 Dr. C. Mas Bermejo Centre for Integrated Adult Dentistry, Plaza de Fuensanta 2, 5° C, 30008 Murcia, Spain; cmasg90@gmail.com; 2 Orthodontics Docent Unit, University Dental Clinic, University of Murcia, 30008 Murcia, Spain; bravo@um.es

**Keywords:** nocturnal hypoxemia, sleep-disordered breathing, oral breathing, upper airway, craniofacial phenotype, cancer

## Abstract

**Background/Objectives:** Human breathing occurs through either the nasal or the oral route. Nasal breathing is the physiological mode of ventilation and contributes to nitric oxide delivery, upper-airway regulation, and efficient pulmonary gas exchange. In contrast, chronic oral breathing has been associated with upper-airway dysfunction, impaired nocturnal oxygenation, and chronic intermittent hypoxia, conditions increasingly associated with cardiovascular, metabolic, respiratory, neurocognitive, and oncological diseases. To determine whether adults with cancer exhibit structural and functional craniofacial characteristics associated with oral breathing patterns and altered nocturnal oxygenation. **Methods:** We conducted an exploratory case–control study including adults with cancer and matched controls. Participants underwent a standardized multidimensional airway assessment comprising symptom evaluation using the STOP-BANG questionnaire, structured clinical examination of upper-airway and orofacial characteristics, craniofacial assessment, cone-beam computed tomography (CBCT), and home respiratory polygraphy to characterize upper-airway phenotype and nocturnal oxygenation patterns. **Results:** Adults with cancer exhibited impaired nocturnal oxygenation despite comparable apnea–hypopnea index values. They also exhibited a higher prevalence of structural and functional upper-airway abnormalities, including restricted tongue mobility, anterior open bite, and reduced maxillary transverse dimensions. **Conclusions:** This exploratory study identifies a distinct upper-airway phenotype in adults with cancer, characterized by craniofacial and functional features associated with impaired nocturnal oxygenation. These findings suggest that upper-airway anatomy and nocturnal oxygenation may contribute to systemic disease vulnerability and warrant further investigation.

## 1. Introduction

Airflow enters the human respiratory system through either the nasal or oral cavity, although nasal breathing represents the physiological route for ventilation. Beyond conducting inspired air, the nose contributes to humidification, filtration, regulation of airway resistance, and endogenous nitric oxide production, which plays an important role in pulmonary vasodilation and ventilation–perfusion matching. Maintenance of stable nocturnal oxygenation therefore depends not only on pulmonary gas exchange itself but also on the structural and functional integrity of the upper airway.

When nasal breathing is impaired because of structural or functional upper-airway obstruction, compensatory oral breathing may develop. Oral breathing has been associated with altered tongue posture, impaired upper-airway mechanics, reduced airway stability during sleep, and an increased susceptibility to sleep-disordered breathing and intermittent hypoxia [1,2]. Craniofacial morphology, tongue mobility, maxillary development, and upper-airway dimensions may further influence airflow dynamics and oxygenation during sleep.

Sleep-related intermittent hypoxia is increasingly recognized as a biologically relevant systemic stressor associated with oxidative stress, endothelial dysfunction, inflammation, autonomic dysregulation, and activation of hypoxia-related molecular pathways. In oncology populations, sleep-disordered breathing and nocturnal hypoxia have been associated with adverse clinical outcomes, including increased cancer incidence, progression, and mortality [3,4]. However, the anatomical and functional airway-related factors potentially contributing to altered nocturnal oxygenation in patients with cancer remain insufficiently explored.

Previous studies have investigated associations between craniofacial dimensions and sleep-disordered breathing in non-cancer populations, including analyses based on CBCT-derived anatomical parameters [1,2,5]. Reduced maxillary dimensions, altered tongue posture, nasal airflow limitation, and restricted tongue mobility have been associated with impaired airway stability and increased susceptibility to intermittent hypoxia. Nevertheless, these studies are limited by heterogeneous methodologies and incomplete characterization of functional airway variables and breathing patterns.

From a physiological perspective, variability in upper-airway stability and breathing behavior may influence nocturnal oxygenation independently of conventional event-based metrics such as the apnea–hypopnea index (AHI). This concept may be particularly relevant in patients with cancer, in whom increased biological vulnerability could amplify the systemic consequences of impaired nocturnal oxygenation.

**Figure 1:**
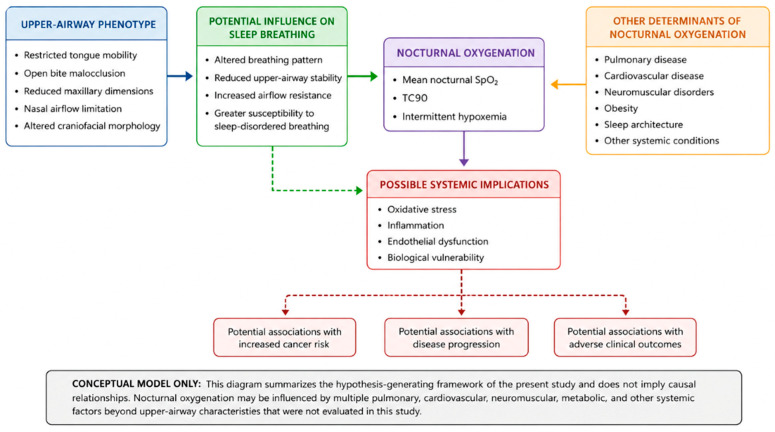
Conceptual framework illustrating the potential relationships between upper-airway phenotype and nocturnal oxygenation in adults with cancer. This diagram summarizes the conceptual rationale underlying the present study and is intended solely as a hypothesis-generating model. Upper-airway anatomical and functional characteristics may contribute to altered breathing patterns and nocturnal oxygenation. However, nocturnal oxygenation is also influenced by multiple pulmonary, cardiovascular, neuromuscular, metabolic, and other systemic factors that were not evaluated in the present study. Accordingly, this figure should not be interpreted as implying causal relationships.

The aim of the present exploratory case–control study was to characterize craniofacial, airway-related, and sleep-breathing features associated with nocturnal oxygenation patterns in adults with cancer compared with matched controls. A major strength of this study is the integration of detailed stomatological, craniofacial, and sleep-related assessments to provide a multidimensional characterization of airway phenotype and oxygenation patterns (Figure 1).

## 2. Materials and Methods

### 2.1. Study Design and Population

We conducted an exploratory observational case–control study between February and November 2025 at two university-affiliated stomatology clinics in Murcia, Spain. In Spain, stomatology is a medical specialty focused on the diagnosis and management of oral, craniofacial, and upper-airway-related conditions.

Adults with histologically confirmed cancer of any type were recruited consecutively as cases. Given the descriptive aim of the study, different cancer types were grouped together. For participants with cancer, only the anatomical site of the primary tumor was recorded. Detailed oncological variables, including cancer stage, treatment history, disease status, and time since diagnosis, were not systematically collected because these variables were outside the primary scope of the study, which focused on airway phenotype and nocturnal oxygenation.

Controls consisted of adults aged 35–85 years without a history of cancer who attended the same clinics for routine oral health complaints and first-time stomatological evaluation. Control participants were recruited consecutively using the same inclusion and exclusion criteria as the cancer group, except for the absence of cancer.

The study followed general methodological principles for case–control research [6,7]. Given the exploratory pilot design, no formal sample size calculation was performed. Therefore, all statistical analyses were considered exploratory and hypothesis-generating.

### 2.2. Eligibility Criteria

Inclusion criteria:

- Age 35–85 years
- Ability to provide informed consent
- Availability for clinical assessment and polygraphy

Exclusion criteria:

- Prior orthognathic or craniofacial surgery
- Congenital craniofacial syndromes
- Cleft lip or palate
- Conditions affecting maxillary structure (e.g., trauma, prosthetics)

Comorbidities, smoking status, medication use, and cancer treatment history were not used as exclusion criteria and were not systematically recorded.

### 2.3. Ethical Approval

This study was conducted in accordance with the Declaration of Helsinki. Ethical approval was obtained from two independent committees: the Research Ethics Committee (CEI) of the University of Murcia, Spain (approval code M10/2023/144; Act 7/2024/CEI, 25 April 2024), and the Ethics Committee for Research with Medicines (CEIm/CEI) of the Hospital General Universitario José María Morales Meseguer, Murcia, Spain (approval code C.P. 2023/097—C.I. EST: 34/24, 16 July 2024). All participants provided written informed consent prior to inclusion in the study.

### 2.4. Instrumentation and Data Collection

Data were collected using standardized clinical protocols in accordance with the guidelines established by the Orthodontic Department, Faculty of Dentistry, University of Murcia.

The assessment protocol included three main components: (1) symptom assessment, (2) clinical examination, and (3) complementary diagnostic investigations.

#### 2.4.1. Symptom Assessment

Subjective symptoms related to sleep-disordered breathing were evaluated using the STOP-BANG questionnaire [8]. This validated eight-item screening tool assesses snoring, daytime tiredness, observed apnoeas, hypertension, body mass index, age, neck circumference, and male sex. The validated Spanish version was administered with the assistance of trained personnel to ensure completeness and accuracy. A score ≥ 3 was considered indicative of increased risk for obstructive sleep apnoea.

#### 2.4.2. Clinical Examination

A comprehensive stomatological and maxillofacial examination was performed in all participants. The assessment included demographic and medical data, oncological diagnosis, craniofacial morphology, upper-airway characteristics, and dental occlusion. Maxillary transverse width and anteroposterior dimensions were evaluated clinically, and malocclusion traits such as open bite, crossbite, and dental crowding were recorded. Tongue mobility was graded using the Kotlow classification of ankyloglossia [2,9], considering both mean grade and the presence of restriction (grade ≥ 1). Tongue indentations were recorded as indirect indicators of altered tongue posture or reduced intraoral space. Nasal valve collapse was assessed using the Von Arx scale, while septal deviation and turbinate hypertrophy were recorded as indicators of nasal airflow limitation. Oropharyngeal space was evaluated using the Mallampati and Friedman classifications, and uvula hypertrophy was also documented.

All examinations were performed using standardized protocols by calibrated clinicians.

#### 2.4.3. Complementary Diagnostic Investigations

- **Sleep polygraphy**

Overnight respiratory polygraphy was performed at home using the ApneaLink™ Air system (ResMed, San Diego, CA, USA). The device recorded nasal airflow through a pressure transducer, thoracoabdominal respiratory effort, snoring, and peripheral oxygen saturation via finger pulse oximetry. Data were analysed using ApneaLink™ Air Software (version 10.00, ResMed) to calculate the apnoea–hypopnoea index (AHI, events/h), mean oxygen saturation (SpO_2_), and cumulative time with oxygen saturation below 90% (TC90, min). For descriptive purposes, nocturnal oxygen desaturation severity was classified according to TC90 as mild (0 min), moderate (1–14 min), severe (15–29 min), and very severe (≥30 min), based on the International Consensus Document on Obstructive Sleep Apnea of the Spanish Sleep Network [10]. Hypopnoeas were scored according to AASM criteria using a 4% oxygen desaturation threshold. An AHI ≥ 5 events/h was considered indicative of sleep-disordered breathing.

- CBCT imaging

Three-dimensional craniofacial analyses were performed in all participants using a KODAK 9500 Cone Beam 3D system (Carestream Dental LLC, Atlanta, GA, USA). Scans were acquired at 90 kV, 10 mA, and a voxel size of 0.2 mm. Image reconstruction and linear measurements were performed using CS 3D Imaging^®^ software (version 8.0.34; Carestream Dental LLC, Atlanta, GA, USA).

Transverse maxillary dimension (AE) and anteroposterior maxillary dimension (AF) were measured according to previously validated anatomical landmarks in the coronal and sagittal planes [6,8,10]. Values below 36 mm for AE and 97 mm for AF were classified as deficient. All measurements were performed by an experienced examiner using standardized anatomical landmarks.

All data were entered into a standardized database for statistical analysis.

### 2.5. Statistical Analysis

Continuous variables were summarized as mean ± standard deviation (SD) or median (interquartile range [IQR]), depending on data distribution. Categorical variables were presented as frequencies and percentages.

Normality of continuous variables was assessed using visual inspection and the Shapiro–Wilk test.

Between-group comparisons were performed using independent-samples t-tests for normally distributed continuous variables and Mann–Whitney U tests for non-normally distributed variables. Categorical variables were compared using Pearson’s chi-square test or Fisher’s exact test when appropriate.

Given the exploratory nature of this pilot study and the limited sample size, no adjustment for multiple comparisons was applied. Therefore, the results should be interpreted as hypothesis-generating rather than confirmatory. Statistical significance was defined as a two-sided *p*-value < 0.05.

All statistical analyses were performed using R (R Foundation for Statistical Computing, Vienna, Austria).

## 3. Results

### 3.1. Participant Characteristics

Sixty-three adults were included in the analysis: 30 participants with cancer and 33 controls without a history of cancer. Mean age was similar between groups (cancer: 63.9 ± 10.9 years; controls: 63.0 ± 8.2 years; *p* = 0.35). Sex distribution did not differ significantly between groups (*p* = 0.56). Categorical body mass index (BMI) distribution did not differ significantly between groups (*p* = 0.14), although severe obesity was more frequent among participants with cancer. Baseline demographic characteristics are summarized in Table 1.

### 3.2. Sleep-Related Symptom Questionnaire (STOP BANG)

The proportion of participants with STOP-BANG scores ≥ 3 was high in both groups and did not differ significantly between participants with cancer and controls (76.7% vs. 66.7%; OR = 1.65, 95% CI 0.54–5.05; *p* = 0.37) (Table 2).

### 3.3. Craniofacial and Maxillary Dimensions

CBCT measurements were obtained in all participants included in the study (30 cancer patients and 33 controls). Mean transverse maxillary width (AE) was significantly smaller in the cancer group compared with controls (30.38 ± 4.1 mm vs. 33.7 ± 2.15 mm; *p* = 0.009). Mean anteroposterior maxillary dimensión (AF) was also lower in the cancer group (87.54 ± 5.6 mm vs. 89.7 ± 6.1 mm), although this difference did not reach statistical significance (*p* = 0.068). CBCT measurements are summarized in Table 3.

### 3.4. Oral and Oropharyngeal Findings

Restricted tongue mobility was significantly more frequent in the cancer group, with a higher proportion of participants presenting moderate-to-severe restriction (Kotlow grade ≥ 3: 76.7% vs. 48.5%; *p* = 0.002). Open bite malocclusion was also more prevalent among cancer participants compared with controls (26.7% vs. 3.0%; *p* = 0.011). Overbite showed a borderline association (23.3% vs. 6.1%; *p* = 0.050), whereas crossbite and dental crowding did not differ significantly between groups.

Tongue indentations, septal deviation, turbinate hypertrophy, nasal valve findings, breathing pattern, Mallampati classification, Friedman classification, and CBCT-derived airway measurements did not differ significantly between groups.

Oral, occlusal, and airway-related findings are summarized in Table 4A–D.

### 3.5. Respiratory Polygraphy Findings

In contrast, the distribution of apnea–hypopnea index (AHI) severity categories did not differ significantly between groups (*p* = 0.238), indicating that the overall severity of sleep-disordered breathing was comparable between cancer patients and controls.

Despite similar AHI severity, nocturnal oxygenation was significantly worse in the cancer group. The proportion of participants classified as having very severe nocturnal oxygen desaturation according to TC90 categories was higher in the cancer group compared with controls (50.0% vs. 18.2%; *p* = 0.047). Likewise, reduced mean nocturnal oxygen saturation according to predefined oxygenation thresholds was more frequent among participants with cancer (76.7% vs. 51.5%; OR = 3.09, 95% CI 1.04–9.17; *p* = 0.038).

Mean nocturnal heart rate was higher in the cancer group (70.98 ± 13.25 bpm vs. 64.30 ± 9.86 bpm; *p* < 0.05). Respiratory polygraphy parameters are summarized in Table 5A–C.

## 4. Discussion

This exploratory case–control study identified a distinct pattern of upper-airway and sleep-related characteristics in adults with cancer. Although cancer participants showed similar STOP-BANG screening results and comparable distributions of apnea–hypopnea index (AHI) severity categories, they exhibited significantly greater impairment in nocturnal oxygenation reflected by a higher prevalence of severe oxygen desaturation and reduced mean nocturnal oxygen saturation. In addition, several craniofacial and functional airway-related characteristics, including restricted tongue mobility, open bite malocclusion, and reduced transverse maxillary dimensions, were more frequent among participants with cancer.

One of the most relevant findings of the present study is the apparent dissociation between conventional event-based measures of sleep-disordered breathing and oxygenation-related outcomes. Despite the absence of significant differences in AHI severity categories, cancer participants demonstrated substantially greater nocturnal oxygen desaturation. This observation is consistent with growing evidence suggesting that AHI alone may not fully capture the physiological consequences of sleep-disordered breathing. Oxygenation metrics such as TC90 and mean nocturnal oxygen saturation may provide complementary information regarding the cumulative biological burden of respiratory disturbances during sleep.

From a physiological perspective, maintaining adequate tissue oxygenation is the primary physiological purpose of the respiratory system. Respiratory physiology involves the integrated interaction of external respiration, pulmonary gas exchange, and internal cellular respiration, all of which depend on sufficient oxygen delivery to sustain mitochondrial ATP production and normal organ function. Consequently, recurrent nocturnal hypoxia may represent a biologically relevant systemic stressor that extends beyond conventional respiratory outcomes. 

In oncology populations, intermittent hypoxia has been associated with activation of hypoxia-inducible pathways, oxidative stress, inflammation, angiogenesis, and tumor-related biological processes [11]. Recent clinical evidence also supports an association between obstructive sleep apnea and the incidence and mortality of urological cancers [12]. Furthermore, recent translational research has strengthened the biological rationale linking obstructive sleep apnea, intermittent hypoxia, and lung cancer through molecular mechanisms that may influence tumor initiation and progression [13]. Long-term clinical follow-up studies also suggest that effective treatment of obstructive sleep apnea may influence cancer-related outcomes, although additional prospective evidence is needed [14]. 

Although the present study was not designed to investigate cancer progression or mechanistic pathways, the observation of impaired nocturnal oxygenation among cancer participants supports further investigation of oxygenation-related variables in this population. The findings should be interpreted as associations rather than evidence of causality. Beyond the present findings, the clinical impact of chronic hypoxemia extends beyond respiratory disease and oncology, having also been associated with cognitive impairment and neurological dysfunction across multiple chronic hypoxic conditions [15]. Recent clinical evidence has also demonstrated that sleep-related hypoxemia is independently associated with poorer survival in patients with non-small cell lung cancer, highlighting the potential prognostic value of nocturnal oxygenation in oncology [16].

From a structural perspective, cancer participants exhibited significantly smaller transverse maxillary dimensions on CBCT assessment. Reduced transverse maxillary dimensions have previously been associated with altered upper-airway anatomy, impaired nasal airflow, and increased susceptibility to sleep-disordered breathing. Although anteroposterior maxillary dimensions did not differ significantly between groups, the consistent trend toward smaller craniofacial dimensions in cancer participants may suggest an anatomical contribution to altered nocturnal oxygenation patterns.

Restricted tongue mobility was also significantly more frequent among cancer participants [17]. Previous studies have suggested that tongue restriction may influence tongue posture, maxillary development, and upper-airway stability. Altered tongue posture may contribute to oral breathing patterns and increased airway collapsibility during sleep. Although the present study cannot establish a causal relationship, the higher prevalence of tongue restriction observed in the cancer group supports the potential relevance of orofacial functional factors in airway phenotype characterization.

Anterior open bite malocclusion was significantly more common among cancer participants. Open bite has been associated with oral breathing, altered neuromuscular balance, and abnormal craniofacial development. Together with restricted tongue mobility and reduced maxillary dimensions, these findings support the concept that structural and functional airway-related characteristics may cluster within a broader airway phenotype associated with altered nocturnal oxygenation.

In contrast, other anatomical variables traditionally associated with upper-airway obstruction, including septal deviation, turbinate hypertrophy, nasal valve findings, Mallampati classification, Friedman classification, breathing pattern, and CBCT-derived airway measurements, were highly prevalent in both groups but did not significantly differentiate cancer participants from controls. These findings suggest that such variables may represent common background characteristics rather than primary discriminative factors within this population.

From a clinical perspective, these results highlight the limitations of relying exclusively on screening tools and AHI-based classification in oncology populations. A more comprehensive assessment incorporating oxygenation metrics and craniofacial evaluation may improve phenotypic characterization and risk stratification. This concept is supported by recent evidence suggesting that nocturnal hypoxemia may be a stronger predictor of colorectal neoplasia than obstructive sleep apnea itself, reinforcing the value of oxygenation-related metrics beyond conventional event-based indices [18].

Identification of structural factors associated with altered airway function, such as restricted tongue mobility, may also have implications for interdisciplinary management approaches involving stomatology, orthodontics, and sleep medicine.

The inclusion of a detailed stomatological assessment may provide additional insights into structural and functional contributors to airway instability that are not routinely captured in standard clinical pathways. This approach allows a more comprehensive characterization of anatomical and functional factors potentially influencing airway stability and nocturnal oxygenation.

This study also has several strengths. All participants underwent a standardized multidimensional assessment including symptom evaluation, detailed stomatological and craniofacial examination, cone-beam computed tomography (CBCT), and home respiratory polygraphy. This comprehensive protocol allowed the simultaneous evaluation of structural, functional, and physiological variables potentially associated with nocturnal oxygenation patterns. 

Future studies should evaluate these findings in larger and more clinically homogeneous populations, incorporating detailed oncological variables and longitudinal follow-up. The incorporation of circulating biomarkers may also help to clarify the biological relationship between sleep-disordered breathing, nocturnal hypoxemia, and cancer progression, providing complementary information to anatomical and physiological phenotyping [19]. Furthermore, recent evidence indicates that episodic hypoxemia may accelerate lung cancer recurrence and mortality, reinforcing the potential clinical significance of nocturnal oxygenation as a prognostic factor [4]. 

These observations are consistent with the growing recognition that comprehensive evaluation of sleep-disordered breathing should extend beyond conventional event-based metrics and include detailed assessment of nocturnal oxygenation and upper-airway characteristics [10].

Such investigations may help clarify whether airway phenotype and nocturnal oxygenation represent clinically relevant markers of physiological vulnerability in patients with cancer and other chronic systemic diseases.

## 5. Limitations

This study has several limitations that should be acknowledged. First, the exploratory case–control design and relatively small sample size limit statistical power, causal interpretation, and generalizability of the findings. Second, the cancer cohort was heterogeneous and included participants with different tumor locations, which may have introduced substantial clinical variability.

Several potentially important confounding variables, including smoking status, alcohol consumption, cardiopulmonary disease, anemia, cachexia, medication use, cancer stage, treatment history, disease status, and time since diagnosis, were not systematically recorded because of the exploratory and pilot-scale nature of the study. These factors may influence sleep-related breathing parameters and nocturnal oxygenation and should therefore be considered when interpreting the findings.

Home respiratory polygraphy was used instead of full polysomnography, and no multivariable modeling or correction for multiple exploratory comparisons was performed. Therefore, the findings should be interpreted as exploratory and hypothesis-generating rather than confirmatory.

Further studies using larger cohorts, more homogeneous cancer populations, standardized oxygenation metrics, longitudinal follow-up, and phenotype-oriented analytical approaches are needed to better characterize the relationship between airway phenotype, nocturnal oxygenation, and systemic physiological vulnerability.

## 6. Conclusions

In this exploratory case–control study, adults with cancer demonstrated altered nocturnal oxygenation despite broadly comparable apnea–hypopnea index (AHI) values and sleep-disordered breathing severity categories compared with controls. Oxygenation-related parameters, including TC90 and mean nocturnal oxygen saturation, appeared to identify differences between groups that were not captured by conventional event-based indices alone.

Structurally, participants with cancer showed a higher prevalence of restricted tongue mobility and anterior open bite malocclusion, suggesting an association between airway-related craniofacial characteristics and altered nocturnal oxygenation patterns. In contrast, other anatomical features, including nasal and oropharyngeal variables, were common across participants but did not significantly differentiate between groups.

These findings support the concept that multidimensional assessment of upper-airway phenotype and nocturnal oxygenation may provide clinically relevant information beyond conventional AHI-based classification in oncology populations.

The integration of oxygenation metrics, stomatological examination, and craniofacial assessment may contribute to a more comprehensive characterization of airway phenotype and physiological vulnerability.

Although causal relationships cannot be established, the results support further investigation of the relationship between airway phenotype, nocturnal oxygenation, and systemic disease processes in larger prospective studies. Such approaches may contribute to improved phenotypic characterization and risk stratification in adults with cancer.

## Figures and Tables

**Table 1:** Baseline demographic characteristics.

Variable	Cancer (*n* = 30)	Control (*n* = 33)	*p*-Value
Age (years), mean ± SD	63.9 ± 10.9	63.0 ± 8.2	0.35
Sex (male)	9 (30.0%)	13 (39.4%)	0.56
**BMI category**			0.14
Normal weight (<25 kg/m^2^)	**7 (17.5%)**	**11 (27.5%)**	Normal weight (<25 kg/m^2^)
Overweight (25–29.9 kg/m^2^)	**18 (45.0%)**	**18 (45.0%)**	Overweight (25–29.9 kg/m^2^)
Obesity (30–39.9 kg/m^2^)	**12 (30.0%)**	**3 (7.5%)**	Obesity (30–39.9 kg/m^2^)
Morbid obesity (≥40 kg/m^2^)	**3 (7.5%)**	**1 (2.5%)**	Morbid obesity (≥40 kg/m^2^)

**Table 2:** STOP-BANG screening risk categories.

Variable	Cancer (*n* = 30)	Control (*n* = 33)	OR (95% CI)	*p*-Value
STOP-BANG ≥ 3	23/30 (76.7%)	22/33 (66.7%)	1.65 (0.54–5.05)	0.37
STOP-BANG < 3	7/30 (23.3%)	11/33 (33.3%)	Reference	—

**Note:** Data are expressed as number/total (percentage). Odds ratios (OR) compare the cancer group with controls; 95% confidence intervals (CI) and *p* values were obtained using Fisher’s exact test.

**Table 3:** Maxillary (CBCT) measurements.

Measurement	Cancer (CBCT Subset)	Control (CBCT Subset)	*p*-Value
Transverse width (AE, mm), mean ± SD	30.38 ± 4.1	33.7 ± 2.15	0.009
Anteroposterior length (AF, mm), mean ± SD	87.54 ± 5.6	89.7 ± 6.1	0.068
AE < 36 mm, *n* (%)	27/30 (90.0%)	25/33 (75.8%)	0.15
AF < 97 mm, *n* (%)	29/30 (96.7%)	29/33 (87.9%)	0.29

**Table 4:** (**A**): Oral and structural findings. (**B**): Occlusal traits. (**C**): Airway and soft findings. (**D**): CBCT airway-related findings.

**(A)**				
**Variable**	**Category**	**Cancer *n*/N (%)**	**Control *n*/N (%)**	***p*** **-value**
**Lingual frenulum (Kotlow 0–5)**	≥3 (restricted)	23/30 (76.7%)	16/33 (48.5%)	**0.002**
	<3	7/30 (23.3%)	17/33 (51.5%)	
**Tongue indentations**	Present	28/30 (93.3%)	29/33 (87.9%)	0.461
	Absent	2/30 (6.7%)	4/33 (12.1%)	
**Dental crowding (maxillary)**	Present	12/30 (40.0%)	12/33 (36.4%)	0.767
	Absent	18/30 (60.0%)	21/33 (63.6%)	
**Dental crowding (mandibular)**	Present	15/30 (50.0%)	14/33 (42.4%)	0.547
	Absent	15/30 (50.0%)	19/33 (57.6%)	
**Edentulism**	Any	23/30 (76.7%)	28/33 (84.8%)	0.104
	None	7/30 (23.3%)	5/33 (15.2%)	
**(B)**				
**Variable**	**Category**	**Cancer *n*/N (%)**	**Control *n*/N (%)**	***p*** **-value**
**Crossbite**	Present	11/30 (36.7%)	14/33 (42.4%)	0.713
	Absent	19/30 (63.3%)	19/33 (57.6%)	
**Open bite**	Present	8/30 (26.7%)	1/33 (3.0%)	**0.011**
	Absent	22/30 (73.3%)	32/33 (97.0%)	
**Overbite**	Present	7/30 (23.3%)	2/33 (6.1%)	**0.050**
	Absent	23/30 (76.7%)	31/33 (93.9%)	
**(C)**				
**Variable**	**Category**	**Cancer *n*/N (%)**	**Control *n*/N (%)**	***p*** **-value**
**Nasal septum deviation**	Present	25/30 (83.3%)	26/33 (78.8%)	0.646
	Absent	5/30 (16.7%)	7/33 (21.2%)	
**Nasal valve (Von Arx)**	Multiple categories	—	—	0.256
**Mallampati (1–4)**	Multiple categories	—	—	0.173
**Friedman classification**	Multiple categories	—	—	0.348
**Breathing pattern**	Mixed predominance	27/30 (90.0%)	29/33 (87.9%)	0.531
**Dry lips**	Present	30/30 (100%)	31/33 (93.9%)	0.171
**(D)**				
**Variable**	**Category**	**Cancer *n*/N (%)**	**Control *n*/N (%)**	***p*** **-value**
**Turbinate hypertrophy (CBCT)**	Present	25/30 (83.3%)	27/33 (81.8%)	0.923
	Absent	5/30 (16.7%)	6/33 (18.2%)	
**Oropharyngeal A–P narrowing**	Narrow	26/30 (86.7%)	28/33 (84.8%)	0.837
	Normal	4/30 (13.3%)	5/33 (15.2%)	

**Note:** Data are presented as number/total (percentage). *p*-values were calculated using chi-square or Fisher’s exact test where appropriate.

**Table 5:** Home respiratory polygraphy.

**(A): Apnea–Hypopnea Index (AHI)**				
**Severity (AHI)**		**Cancer**	**Control**	***p*** **-value**
Normal (<5)		16/30	24/33	**0.238**
Mild (5–14)		6/30	5/33	
Moderate (15–29)		3/30	3/33	
Severe (≥30)		5/30	1/33	
**(B): Oxygen desaturation (TC90%)**				
**TC90% category**		**Cancer *n*/N (%)**	**Control *n*/N (%)**	***p*** **-value**
Mild		3/30 (10.0%)	8/33 (24.2%)	
Moderate		10/30 (33.3%)	14/33 (42.4%)	
Severe		2/30 (6.7%)	5/33 (15.2%)	
Very severe		15/30 (50.0%)	6/33 (18.2%)	**0.047**
**(C): Mean nocturnal oxygen saturation categories**				
**Variable**	**Cancer *n*/N (%)**	**Control *n*/N (%)**	***p*** **-value**	**OR (95% CI)**
**Hypoxia**	23/30 (76.7%)	17/33 (51.5%)	**0.038**	**3.09 (1.04–9.17)**
**No hypoxia**	7/30 (23.3%)	16/33 (48.5%)	—	—

*p* values were calculated using the Pearson χ^2^ test.

## Data Availability

The data presented in this study are available from the corresponding author upon reasonable request.
